# A Qualitative Study of Vape Shop Operators' Perceptions of Risks and Benefits of E-Cigarette Use and Attitude Toward Their Potential Regulation by the US Food and Drug Administration, Florida, Georgia, South Carolina, or North Carolina, 2015

**DOI:** 10.5888/pcd13.160071

**Published:** 2016-05-19

**Authors:** Pratibha Nayak, Catherine B. Kemp, Pamela Redmon

**Affiliations:** Author Affiliations: Catherine B. Kemp^,^ Pamela Redmon, Georgia State University Tobacco Center of Regulatory Science, School of Public Health, Georgia State University, Atlanta, Georgia.

## Abstract

**Introduction:**

Approximately 8,500 vape shops in the United States sell a variety of electronic nicotine delivery systems (ENDS). This study examined vape shop operators’ perceptions of benefits and risk of ENDS use, what they perceive to be the reasons for ENDS use, their source of product information, what information they shared with customers, and the impact of existing and future regulation of ENDS on its use and on their business.

**Methods:**

We conducted qualitative interviews with 20 vape shop operators located in Florida, Georgia, South Carolina, and North Carolina in spring 2015. A semi-structured interview guide was used, and interviews were audio-recorded and transcribed verbatim. The transcripts were analyzed using NVIVO software.

**Result:**

Vape shop owners perceived ENDS to be less harmful and more economical than conventional cigarettes and indicated that most of their customers used ENDS as a smoking cessation tool. Most owners were former smokers and used ENDS to quit. Shop owners relied on their personal experiences and the Internet for information, and shared information with customers at point of sale by using the shop’s website and social media. Most expressed concern that complying with potential regulations, including banning flavors or tax increases, would jeopardize their business. Some felt that ENDS should not be regulated as tobacco products and felt that big tobacco was behind these proposed regulations. Most owners supported age restrictions and quality controls for e-liquid.

**Conclusion:**

Vape shop owners are in a unique position to serve as frontline consumer educators. Interventions should focus on providing them with current information on benefits and risks of ENDS and information on national, state, and local regulations and compliance requirements.

## Introduction

Electronic nicotine delivery systems (ENDS) entered the US market little less than a decade ago, and sales were projected to reach $3.5 billion by the end of 2015 (1). These products are popular among current and former smokers who perceive them as smoking reduction or cessation aids (2,3). ENDS are battery-powered heating coils that aerosolize liquid containing propylene glycol or vegetable glycerin mixed with flavorings and nicotine (4). ENDS are broadly classified into 2 categories: closed systems (cigalikes), and open systems (eg, vape pens, vaporizers, vapes, tanks, eGo style) (5,6). ENDS are sold principally in stores known as vape shops (7).

Currently, no federal regulations govern ENDS or e-liquids (8), and their advertising is not restricted (9). In 2014, the US Food and Drug Administration (FDA) announced its intention to extend its authority to regulate additional tobacco products such as ENDS (8,10). The anticipated FDA rule will likely affect ENDS products and vape shop operations.

Vape shop operators play a significant role in marketing ENDS and use strategies similar to tobacco industry strategies, such as giving away free samples of e-liquids, sponsoring events, and using marketing strategies to increase acceptability of ENDS (11). Shop operators help customers determine e-liquid nicotine levels and sample flavorings, and they provide information on device use and product safety (12). Their product knowledge and perceptions of risk and benefits of ENDS use may affect the information they share with customers (13). This study explored vape shop operators' perceptions of benefits and risks of ENDS, their customers’ reasons for use and sources of information on vapes, and what information operators shared with customers. It also assessed operators’ perceptions of potential future FDA regulation of these products on its use and on their business.

## Methods

In spring 2015, semi-structured, in-depth telephone interviews were conducted with vape shop operators located in 4 states in the southeastern United States: Florida, Georgia, North Carolina, and South Carolina. A previous study (14) indicated that thematic saturation could be reached after 12 interviews; on the basis of this information, we decided to interview at least 20 vape shop operators to ensure thematic saturation and to account for any variability in data collection across the 4 states, intending to interview more participants if more data were needed. Five zip codes per state were randomly selected by using Google Maps, and Google searches for vape stores were conducted within the selected zip code. The operator of the first shop listed in the Google search from each zip code in each state was contacted by telephone to participate in the study. To be eligible for the study, participants had to operate a vape shop, had to be older than 18 years by self-report, and had to speak English. Overall, operators of 68 vape shops were contacted to participate in this study; 40 did not respond (ie, could not be reached by telephone, did not return calls after 3 attempts, were not available, or were not available when we called back at their designated time), 7 refused to participate, and 1 was ineligible. Twenty of the 27 vape shop operators identified as eligible were interviewed (74% participation rate). Interviews were conducted by trained interviewers, lasted for approximately 20 minutes, and were audio-recorded and transcribed verbatim. Participants were offered $50 as remuneration for their time. The institutional review board of Georgia State University approved this study.

Information about the operators’ sex and whether they were the owner or manager of the shop were collected at the time of recruitment. Information regarding operators' smoking and vaping status was coded during the data analysis. Sociodemographic data related to each vape shop’s location and business characteristics were collected through public sources. This data consisted of rural-urban continuum information (15), average income relative to federal poverty guidelines (16,17), post-secondary school(s) within proximity to the shop’s location (18), online presence of the shop, and whether the business had single or multiple locations. Additional details on the measures used to describe sociodemographic characteristics of the vape stores studied are in Table 1.

**Table 1:** Sociodemographic Characteristics of Operators of 20 Vape Shops in Florida, Georgia, South Carolina, or North Carolina, 2015

Study Participant Characteristics	N
**Shop owner or co-owner**	
Male	15
**Number of Stores Owned**	
Single store	14
Multiple stores	6
**Referred to personal use of vaping device**	
As a means of smoking cessation	13
As independent of smoking history	4
Not available	3
**Web profile **	
Facebook page	18
Yelp page	20
Store website	17
Online store	11
**Geographic location**	
Within 5 miles of college campus^a^	14
County in metro area of >1 million population^b^	6
County in metro area of 250,000–1 million population^b^	11
County in metro area of <250,000 population^b^	2
County with an urban population of >20,000, not adjacent to a metro area^b^	1
Region with median annual income 1–2 times US federal poverty guidelines (FPG)^c^	1
Region with median annual income 2–3 times FPG^c^	10
Region with median annual income 3–4 times FPG^c^	6
Region with median annual income >4 times FPG^c^	3

a Source US Department of Education, Institute of Education Sciences, National Center for Education Statistics (http://nces.ed.gov/globallocator/).

b Source: US Census Bureau (http://www.ers.usda.gov/data-products/rural-urban-continuum-codes/documentation.aspx).

c Median income of zip code where shop is located relative to 2013 Federal Poverty Guideline (FPG) for a family of 4. The 2013 FPG for a family of 4 was $23,550. Source: US Department of Health and Human Services 2013 Poverty Guidelines (http://aspe.hhs.gov/poverty/13poverty.cfm#thresholds). Accessed August 5, 2015.

We developed a semi-structured interview guide and pilot-tested it with 2 vape shop operators to assess feasibility and language and to ensure that the questions and probes generated quality data. The guide was revised on the basis of pilot test results. The semi-structured interview guide explored the following topics: perceptions of benefits and risks associated with ENDS, reasons customers used ENDS, vape shop operators’ sources of information, ENDS information shared with customers, operators’ information needs, current knowledge of ENDS regulations, and perceptions of the potential impact of future regulations on the ENDS industry.

We transcribed all recorded interviews and used NVIVO (QSR International) analytical software to analyze data. To identify emerging themes, 3 transcripts were selected and analyzed by each author independently. Recurrent themes were identified and assigned codes. A master list was created comprising all codes, and the remaining interviews were double-coded. The interrater reliability was 0.8. Discrepancies were resolved by consensus among the authors. Theme saturation was reached after analysis of 8 to 10 transcripts.

## Results

Fifteen of the 20 participants were male, and all were vape shop owners or co-owners (referred to hereafter as “owners”). Most participants stated they became involved in the industry because of their personal vaping experiences as a means of smoking cessation. Most vape shops were located within 5 miles of at least one college or other post-secondary campus. Almost all shops were located in metropolitan areas with populations of 250,000 or more and in zip codes in which the average income for a family of 4 was at least twice that of the federal poverty guidelines. All shops had some online presence to promote their businesses and to share product information (Table 1).

Vape shop owners were asked to describe their perceptions of the benefits of ENDS, and most owners said their health had improved after substituting vaping for smoking. Examples of their comments were, “I just feel more motivated,” “Don’t cough as much,” and “Everything tastes better.” Most owners believed that ENDS were less harmful than smoking tobacco. Shop owners also mentioned cosmetic benefits of ENDS use, such as lack of teeth staining and unpleasant odors. Other benefits reported were that ENDS were less likely to cause fires and were safer than traditional cigarettes. Owners indicated that ENDS were cheaper than cigarettes and that cost was an important motivator for customers to switch to ENDS. They further elaborated that although the device required an upfront cost, vaping was more economical than cigarettes in the long run (Table 2).

**Table 2:** Themes and Comments Regarding Perceived Benefits and Risks Associated With Use of Electronic Nicotine Delivery Systems (ENDS) and Reasons for Using ENDS, Consumers at Point of Sale in 20 Vape Shops in Florida, Georgia, South Carolina, or North Carolina, 2015

Themes	Comments	n
Benefits of using ENDS		
Health	“The benefits of using an e-cigarette is – before the e-cigarettes came I smoked two packs a day. I couldn’t climb a set of stairs to save my life; I just stayed out of breath.” “Since I’ve started vaping I smell that litter box smell! So you know, my food tastes better too.”	16
Cessation	“It provides a good alternative to people to cut back on smoking or quit and still provide them with the same satisfaction they get when they’re smoking.” “Just being able to quit without some of the hassle. I mean, a lot of people issue is the mouth to hand coordination that goes along with smoking.”	16
Less harmful than smoking	“But nicotine itself hasn't really shown harmful products as much as the tar and stuff has inside of the cigarettes itself.” “Basically you are cutting out combustion from cigarettes and getting rid of the vast majority of harm that is caused from traditional analog smoking.”	12
Cosmetic	“So you don’t have that smell lingering around. It doesn’t discolor your teeth like cigarettes do.”	6
Risk of using ENDS		
None	“In my opinion there are none” “I personally haven't experienced any negative results of electronic cigarettes.”	17
Health	“And they’ll break out, and the breakout looks like a little rash. And then they find they’re allergic to propylene glycol.” “Some say sometimes that it may irritate their throat.”	9
Uncertain	“Unfortunately, that's not something we entirely know yet, I mean, it's such a new industry. As of right now, that's such a hard question for me to answer because personally I don't see a ton that are – that we know of right now. But we don't know the long-term use risks of this product yet because it is so new.”	8

Vape shop owners’ perception of risks of ENDS was explored, and almost all perceived that ENDS had no health risks; one owner commented “In my opinion there are none.” They indicated they rarely received reports of vape-related harm from their customers. Most said they considered the risk of ENDS as primarily safety concerns related to inappropriate handling or use of the vape device. Some noted that fires could occur from battery explosion, although they felt this was very rare. Some acknowledged that some e-liquids were produced in unclean environments, and some indicated uncertainty of the risk associated with using poorly produced products. Some owners reported incidents of vape smokers being allergic to ingredients in e-liquids. Some also expressed reservations because the long-term health risks of using ENDS are unknown (Table 2).

All vape shop owners said their customers used ENDS to help them quit smoking or to reduce the number of cigarettes they smoked. They indicated that some cigarette smokers continued to use both products. Many owners reported their customers cited lifestyle factors, including health and disease conditions, as motivators to switch from smoking to vaping. In addition, the owners spoke of vaping as a hobby: “Now it's just a hobby, it's something to build. It's a whole other community itself,” and “Some people . . . they don't want to smoke cigarettes, so they can smoke this as a social type of thing.” Several said that ENDS offered alternative ways to access nicotine in locations where smoking was prohibited.

Vape shop owners indicated they relied on personal experiences in using vape products and talks with distributors or the manufacturers of the products to learn more about ENDS. Owners also mentioned conducting Internet research: “The Internet mostly. You meet people that’s been in it for a long time. You let them know that you’re trying to start a business, and they’ll help you find the place where you can get some juice, and certified, and FDA approved.” They reported searching relevant websites, online forums, and social media for ENDS-related information. Several also reported talking with other vape shop owners to obtain information on product lines, pricing, and the business aspects of selling ENDS. Some indicated they read scientific and medical research journals for information on the health effects of ENDS.

When asked about the types of information they shared with customers, vape shop owners reported they provided instructions on ENDS use and helped customers select nicotine levels and e-liquid flavors. They also indicated they disseminated general information, discussed benefits of ENDS, and shared website references such as FDA.gov, e-cigarette forums, industry sites, Facebook, and those presenting research findings. Most presented information to their customers verbally, but some provided printed materials, such as flyers and pamphlets. One participant stated, “I encourage them to go to the FDA.gov. FDA.gov has plenty of research on there, which most people trust the FDA.gov . . . We do have some literature we give them.” Some used their business website and Facebook page to share relevant product-related information.

Vape shop owners were asked to describe information that would be beneficial for them. Many felt they needed more information on long-term health effects and health benefits related to ENDS use. Responses included: “I would like to have more [health] information that I could tell them to go and look at.” and “There are studies all over the place [but] we don’t know the long term effects.” They indicated that having information on ENDS devices, including device components and e-liquid product safety would be helpful. They also indicated that a statement from the medical community would give them confidence in the reliability of information, and an FDA warning statement related to the use of ENDS would be useful. An owner stated, “It would be great if there were maybe some kind of official information sites for dealing with nothing but electronic cigarettes and safety, safety precautions, and stuff of that nature.”

Most owners described ways in which the sale or use of ENDS products was being regulated by their state or local jurisdictions. Most owners stated that ENDS were not currently federally regulated: “There is no approved regulation at this point and time, so we are all basically self-regulating.” However, a few erroneously stated that e-liquids are FDA regulated: “Everything that is in the juice is already FDA approved.” A few described the devices as being federally regulated: “I will say that the regulated devices definitely give you a good wealth of information.” Some owners expressed concern that development of federal regulation of their industry was being influenced by a desire to protect the tobacco industry: “I believe tobacco has a lot of influence on what’s being done and how it’s being reported. That really kind of bothers me.”

The vape shop owners’ opinions of potential future regulation of the vaping industry were explored, and almost all owners were opposed to at least one specific area of ENDS regulation. Most owners opposed banning flavors in e-juice, and the most frequently cited reason for this opposition was that flavorings were no more enticing to children than flavored alcohol. Many owners described self-imposed quality control practices, which eliminated the need for government-imposed controls: “All the bottles I carry have child safety locks, caps on tops of them.” Several owners felt that ENDS should not be regulated as tobacco products, and some expressed opposition to imposing special taxes on their industry. In addition, several felt regulation was unnecessary because they perceive the products to be safe (Table 3).

**Table 3:** Themes and Comments of 20 Vape Shop Operators Regarding Support for Regulation of Electronic Nicotine Delivery Systems, Florida, Georgia, South Carolina, or North Carolina, 2015

Support Regulation	Policy	Comment	
Yes	E-juice safety and quality control	“I do think that the FDA should have a look into how these juices are being made. Just because I do see some of them and they're being made in bathrooms or they're being made in very unhygienic places.”	10
	Age restrictions	“I do like the fact that the FDA, if they’re going to regulate something, there needs to be an age on it because there are a lot of kids that are doing it.”	9
	General	“It should be regulated in some fashion. I think they are trying to over regulate it because they don’t know.”	5
	Device safety	“I think it’s a good idea if the FDA regulates...the production of devices themselves to make sure that they’re safe for the customer.”	3

No	Flavors	“You can walk into any ABC shop there is flavored vodka and there is flavored this or that, but they don't say it is enticing to children because it is regulated.”	13
	Already self-regulating	“All the bottles I carry have child safety locks, caps on tops of them. They have warning labels, visible warning labels. I deal with manufacturers who are already using ISL labs.”	9
	Taxes	“ It makes me think that we, the industry, has come up with a better solution, a healthier solution, and a cheaper solution, and the government wants to be able to tax it, and get their money off of it, like they do cigarettes.”	5
	Not as tobacco product	“I agree that there needs to be some regulations, but we don't want to be grouped in with tobacco industry.”	5
	General	“I’ve been involved with that for a good while now and the regulations that the FDA is trying to pass is totally absurd.”	4

Abbreviation: FDA, Food and Drug Administration.

Many owners supported a few areas of FDA regulation, including regulations that would improve e-juice safety and quality control, and most favored age restrictions preventing children and adolescents from accessing or using ENDS products. Some expressed general support of ENDS regulations, and several supported taxing the products, regulating where they could be sold, and imposing device safety requirements (Table 3).

Vape shop owners were asked their opinions of how future federal regulations might affect their businesses specifically and the ENDS industry in general. Although some felt federal regulations would result in safer products (“Make sure that everything is safe”), most predicted that regulations would negatively affect or eliminate their ability to stay in business. Many predicted that regulations would result in increased cigarette smoking, and some felt consumers would obtain their vaping supplies from less reputable sources. Some also felt federal regulations would result in increased costs to consumers, and several expressed concern that regulations would adversely affect consumers’ perceptions of vaping as a safe alternative to smoking. Despite these expressed concerns, several shop owners said regulation would generally be positive for the industry, and several felt it would legitimize vaping products. (Table 4).

**Table 4:** Comments Regarding 20 Vape Shop Operators’ Support or Nonsupport for Regulation by Food and Drug Administration of Electronic Nicotine Delivery Systems, Florida, Georgia, South Carolina, or North Carolina, 2015

Perceptions of the Likely Effects of Food and Drug Administration (FDA) Regulation			n
Opinion	Rationale	Comment	
Negative	Affect or eliminate viability of business	“Just on the flavors alone would completely kill half the industry.” “It would devastate it.”	15
	Number of smokers will increase	“I think everyone who would, who has switched, and just uses the fruitier flavors and all that would go back to smoking traditional cigarettes.” “Eventually you’ll get to the point where it might be more expensive to use e-liquids than it would to smoke cigarettes and people will go back to smoking cigarettes!”	10
	Users will purchase online or from less reputable sources	“You are going to have vaping here in the hands of people like convenience stores who don't know what they are talking about and will sell to anybody.” “I would think it would actually make it worse. Then you would have a lot of people that are not controlled and not doing things correctly.”	5
	Consumers’ costs will increase	“It’s also harmful to the people who can’t really afford to smoke cigarettes. You figure a person that spends $200-$300 a month on cigarettes can do an e-cigarette for $30 a month.” “I think people would be less inclined to purchase an e-cigarette if it costs comparable to cigarettes.”	5

Positive	Better, safer products for consumers	“I believe there needs to be regulation to put safety out there for everybody and make sure that we are all doing the right things.” “Well we need to make sure that everything is safe and the companies that are making this liquid and the devices themselves are reputable companies. That is important. You can't trust everyone to look out for the consumer.”	6
	Generally good for vaping industry	“We’re pretty supportive of regulations. I mean, we’re willing to mend and mold whatever we need to in our store to fit regulation standards.”	4

## Discussion

Awareness and popularity of ENDS have increased rapidly over the last decade (19), and sales of ENDS in vape shops accounted for $1.2 billion of the estimated $3.5 billion ENDS sales by the end of 2015(1). ENDS are not currently regulated in the United States (4,20), and the health risks and benefits are not yet well established (21). This study found that vape shop owners perceived many more benefits than risks related to ENDS use and stated that vaping was safer and healthier than cigarette smoking. The primary benefits cited by vape shop owners were related to health, and specifically to improved health after using the vape devices to quit smoking. They also stated that most of their customers used ENDS to help them quit smoking or reduce the number of cigarettes smoked. Similar to our study findings, vape store retailers reported that they reduced cigarette smoking by using ENDS and that using e-cigarettes helped them quit cigarette smoking (22). Although ENDS have been touted as cessation devices by consumer groups (23,24) and described by vape owners as effective cessation tools, the evidence that ENDS use leads to cessation is inconclusive (25). Most owners did not perceive health risks related to ENDS and indicated that any associated risks were primarily due to consumer misuse. Some people in the US public health community expressed concern that ENDS will increase nicotine addiction and renormalize cigarette smoking (26,27), but vape shop owners in this study did not mention these issues.

The owners interviewed relied on a variety of information sources (eg, personal experience, manufacturer information, the Internet, other vape shop operators) for their product information. They used their knowledge to advise customers on e-liquids and nicotine level selections, health benefits, and product use. The owners indicated they needed more information on ENDS, particularly their long-term health effects and health benefits. A recent study also found that owners relied on their own experiences with ENDS, lacked the knowledge and skills to synthesize scientific research findings, and had the potential to disseminate inaccurate information to the consumers (13). Interventions aimed at creating and disseminating factual information on ENDS risks and benefits for vape shop operators and other point-of-sale providers is needed. Receiving timely, relevant, and current scientific information that is easy to understand and disseminate could arm these front-line information sources with the necessary tools to educate their customers.

The FDA’s proposed rule would require manufacturers to register with the agency and submit all new products for approval by the FDA (10). Requirements would include reporting products and ingredients, applying health warnings to product packaging, and restricting claims to those supported by evidence. Product sampling, and age at purchase would be restricted (8). Many state and local jurisdictions impose controls (eg, restricting consumer age at sale, regulating how ENDS products are packaged and sold, specifying where the devices can be used) (28). Many vape shop operators knew that FDA did not regulate ENDS. Vape shop operators expressed concern over proposed rules, and almost all opposed at least one area of proposed regulation. Most opposed banning e-liquid flavors, and many felt there was no need for FDA regulations because of self-regulating practices and perceptions about the products’ safety. Although most owners commented they opposed FDA regulating ENDS and expressed concern that it would negatively impact their businesses and increase smoking, some operators were in favor of e-liquid and device quality control measures and age restrictions.

Vape shop owners have misinformation about components used in e-liquids and perceive them to be safe. They may be assuming propylene glycol, glycerin, and food flavorings are safe because the substances meet the FDA standard of “generally recognized as safe” (GRAS). However, GRAS standards apply to additives in foods that are safe for oral consumption; the standards do not hold true for inhalation. This misperception provides an opportunity to educate vape shop owners and the consumers.

The FDA recognizes the important role of tobacco product retailers, and created a website devoted to educating them on current regulations on tobacco products (29). If the FDA deeming rule is finalized, a website and Facebook page created specifically for ENDS retailers could present information about resources; almost all vape shop operators interviewed mentioned they used the Internet as a source for ENDS information. Information for vape shop owners could be shared via fact sheets, webinars, training videos, searchable databases, “frequently asked questions,” and other relevant guidance documents similar to information listed on the current FDA retailers’ website (29).

This study has some limitations. We did not collect data on the sociodemographic characteristics of vape store operators, and an owner’s sociodemographic characteristics may influence their perceptions of benefits and risks of ENDS as well as the sources they use to obtain information about ENDS. The geographical focus for this study was limited to four states in the southeastern United States and may not be generalizable to other US regions. Because of funding constraints, the geographic locations of our study were limited to 4 states. Google searches and mapping tools were used to identify vape stores because of Google’s wide reach and ability to generate relevant results and verifiability. However, using one search engine may have limited the pool of vape shops sampled. A recently published article provides a comprehensive online search method for identifying vape stores, and this and other methods could be considered in future studies (30). In addition, since the cities in the 4 states were randomly selected, vape shops in small- or medium-sized cities were less likely to be selected. We selected vape stores that were listed first on our search results. It is possible that the vape shops selected paid for advertising or used other marketing strategies to have their businesses listed first on Google search results and on Google maps showing where the stores were located.

The results of this study provided important information on vape operators’ perceptions of risks, benefits, information sources, and information needs, and on their attitudes toward proposed FDA regulations related to ENDS. Given the current uncertainty of the health benefits and long-term risks, it will be important to communicate scientific information on ENDS in lay language to vape store operators using modes of communication such as Facebook to reduce the chance of store operators sharing misinformation with consumers. This study also indicated a need to provide educational resources on FDA regulations and compliance requirements pertaining to ENDS if the proposed deeming rule becomes final.
